# Development of heat-shock resistance in *Legionella pneumophila* modeled by experimental evolution

**DOI:** 10.1128/aem.00666-23

**Published:** 2023-09-28

**Authors:** Jeffrey Liang, Gillian Cameron, Sébastien P. Faucher

**Affiliations:** 1 Department of Natural Resource Sciences, McGill University, Sainte-Anne-de-Bellevue, Québec, Canada; 2 Centre de Recherche en Infectiologie Porcine et Avicole (CRIPA), Faculté de Médecine Vétérinaire, Université de Montréal, Saint-Hyacinthe, Québec, Canada; Centers for Disease Control and Prevention, Atlanta, Georgia, USA

**Keywords:** *Legionella pneumophila*, hot water distribution systems, adaptive laboratory evolution, experimental evolution, heat resistance, pasteurization

## Abstract

Because it can grow in buildings with complex hot water distribution systems (HWDS), healthcare facilities recognize the waterborne bacterium *Legionella pneumophila* as a major nosocomial infection threat and often try to clear the systems with a pasteurization process known as superheat-and-flush. After this treatment, many facilities find that the contaminating populations slowly recover, suggesting the possibility of *in situ* evolution favoring increased survival in high-temperature conditions. To mimic this process in a controlled environment, an adaptive laboratory evolution model was used to select a wild-type strain of *L. pneumophila* for survival to transient exposures to temperatures characteristic of routine hot water use or failed pasteurization processes in HWDS. Over their evolution, these populations became insensitive to exposure to 55°C and developed the ability to survive short exposures to 59°C heat shock. Heat-adapted lineages maintained a higher expression of heat-shock genes during low-temperature incubation in freshwater, suggesting a pre-adaptation to heat stress. Although there were distinct mutation profiles in each of the heat-adapted lineages, each acquired multiple mutations in the DnaJ/DnaK/ClpB disaggregase complex, as well as mutations in chaperone *htpG* and protease *clpX*. These mutations were specific to heat-shock survival and were not seen in control lineages included in the experimental model without exposure to heat shock. This study supports *in situ* observations of adaptation to heat stress and demonstrates the potential of *L. pneumophila* to develop resistance to control measures.

**IMPORTANCE:**

As a bacterium that thrives in warm water ecosystems, *Legionella pneumophila* is a key factor motivating regulations on hot water systems. Two major measures to control *Legionella* are high circulating temperatures intended to curtail growth and the use of superheat-and-flush pasteurization processes to eliminate established populations. Facilities often suffer recolonization of their hot water systems; hospitals are particularly at risk due to the severe nosocomial pneumoniae caused by *Legionella*. To understand these long-term survivors, we have used an adaptive laboratory evolution model to replicate this process. We find major differences between the mutational profiles of heat-adapted and heat-naïve *L. pneumophila* populations including mutations in major heat-shock genes like chaperones and proteases. This model demonstrates that well-validated treatment protocols are needed to clear contaminated systems and—in an analog to antibiotic resistance—the importance of complete eradication of the resident population to prevent selection for more persistent bacteria.

## INTRODUCTION

As an obligate intracellular pathogen, the bacterium *Legionella pneumophila* relies on infection of eukaryotic cells in order to acquire nutrients and replicate (1). Though it usually lives in freshwater ecosystems where it targets free-living eukaryotic hosts from diverse clades, *L. pneumophila* is now also known to infect humans where it targets alveolar macrophages (2). Intracellular growth is mediated by the Icm/Dot Type IVb secretion system which translocates bacterial effector proteins into host cells (2). *L. pneumophila* contamination of human-built infrastructure favors infection by aerosolizing the bacteria and facilitating its travel into the lungs (2, 3). Globally, *L. pneumophila* is the main cause of Legionnaires’ disease, a life-threatening pneumonia whose severity and under-diagnosis have led to its high overall case-fatality rate, estimated at between 5% and 10%, with even higher death rates for nosocomial cases (4 – 6). Early suspicions focused on dispersal through contaminated cooling tower emissions (7, 8), but *L. pneumophila* has since been found in diverse engineered water systems, including in whirlpool spas, decorative fountains, and—significantly for nosocomial infection—in hospital plumbing systems (9). Hospitals are particularly at risk both because they must balance high water temperatures against the risks of scalding and because they often house immunocompromised patients who are particularly threatened by exposure to recurrent bacterial contaminants (10, 11).

The engineering of water systems has a major impact on the growth potential of *L. pneumophila* (12, 13). Large buildings with complex plumbing systems are particularly vulnerable to contamination, and studies have shown that multiple clades of *L. pneumophila* can stably co-exist in these systems (14). As a mesophilic bacterium which replicates best between 20°C and 45°C (15), *L. pneumophila* favors colonization of hot water distribution systems. Deprecated fixtures, as well as the improper design of plumbing networks or incorrect estimation of water demand, can lead to stagnation and the existence of dead legs. These regions of low water flow allow scale and biofilms to accumulate—physical structures that can physically protect *Legionella* from exposure to disinfectants (16, 17). Policies that decrease water temperatures in favor of energy savings can result in tepid temperatures that favor the growth *L. pneumophila* and of its eukaryotic hosts (18, 19). Evidence from pilot-scale studies hs suggested that the age and composition of biofilms and their associated microbiota improve the survival of *Legionella* spp. under otherwise hostile conditions of heat or biocide exposure (17, 20, 21). Eukaryotic members of the drinking water microbiome support the biphasic lifestyle of *L. pneumophila* as the nutrient-replete hosts of replicative stage *L. pneumophila*, with the depletion of cellular resources triggering a switch into a stress-hardy mature infectious form (22, 23). Certain free-living amoeba, such as *Vermamoeba vermiformis*, can also adopt an encysted form (24, 25) which is implicated in the preservation of *L. pneumophila* through periods of high stress.

Along with chemical treatment via chlorination or copper-silver ionization, the maintenance of high temperatures along the length of a plumbing system from the water heater through to the terminal outlets is a critical control measure against *L. pneumophila* (26). Remedial high-temperature pasteurization processes, collectively termed superheat-and-flush, aim to push hot water temperatures above what *L. pneumophila* can tolerate (27, 28). The implementation of these strategies can differ depending on the specific architecture of the hot water distribution system, but they aim to raise temperatures throughout the entire system through to the distal outlets. Though remedial pasteurization can be effective at the short-term clearance of *Legionella*, longitudinal studies have shown that re-contamination is common over month- or year-long time scales (29 – 31). It has been reported that repeated use of high-temperature pasteurization to disinfect these water systems can instead lead to the local evolution of more heat resistant *L. pneumophila* populations, like the familiar phenomenon of antibiotic tolerance (26, 28, 29).

In bacteria, heat stress can damage several vital subcomponents of the cell by causing membrane depolarization and damage, destabilization of the nucleoid, and the inactivation and aggregation of vital enzymes and structural proteins (32 – 34). Bacteria have developed a sophisticated heat-shock response, centralized through the alternative sigma factor RpoH that redirects transcription toward stress resistance by driving the production of heat-shock proteins (35, 36). These heat-shock proteins buffer the toxic effects of misfolded and aggregated proteins in the cell—either by promoting refolding or by targeting them for destruction (37, 38). These proteins are broadly conserved among bacterial species and include proteases as well as chaperones, such as HtpG and DnaK, and their co-chaperone proteins, such as DnaJ and GrpE [reviewed in reference (39)]. After the thermal shock is resolved, these proteins are also involved in a negative feedback loop targeting RpoH for destruction by FtsH, a membrane-bound protease (40, 41). This organization is conserved in *L. pneumophila*, with five heat-shock proteins identified in radiolabeling studies (42) and many more annotated from the published genetic sequence (43, 44). Heat-shock proteins are also involved in the early pathogenesis of *L. pneumophila* by promoting the internalization of the bacterium by the host cell and reorganizing host trafficking processes (45 – 47). The LetAS and CpxRA two-component systems are also known to influence heat-shock survival, likely mediated through their regulation of life phase switching during growth and starvation (48, 49).

Adaptive laboratory evolution is an experimental concept that manipulates a chosen organism in a laboratory setting to providing insights into complicated phenomena occurring in the wild (50, 51). To simulate long-term exposure to high temperatures in plumbing systems, we can depend on the short generation times and the relative ease of culture and storage of *L. pneumophila* in laboratory conditions to study the resulting evolution in a controlled and repeatable process. Adaptive laboratory evolution has been applied in numerous bacterial and non-bacterial species to study the trajectories and repeatability of evolutionary responses without *a priori* expectations on the loci which will be targeted (52, 53). Prior studies have used adaptive laboratory evolution to adapt *Escherichia coli* to growth in stably high-temperature or low pH conditions, as well as to survive acute transient stress from ionizing radiation (54 – 56). Notable adaptive laboratory evolution experiments conducted with *L. pneumophila* have been used to study resistance to fluoroquinolone and macrolide antibiotics (57, 58) and adaptation to mouse macrophages (59). As heat stress non-specifically challenges multiple components of the bacterial cell, adaptive laboratory evolution makes an appropriate protocol to study the range of mutations driving the increasing heat tolerance of *L. pneumophila* populations observed in hot water systems.

To bridge the gap between *in situ* observations of *L. pneumophila*’s adaptation to pasteurization and an understanding of the underlying genetic mechanisms, we tailored an adaptive laboratory evolution study design to investigate the evolutionary paths that the environmental pathogen can take to develop a tolerance of heat shock. The aim was to document the genetic changes that surviving population would develop under transient exposure to high temperatures, simulating failed pasteurizations or bursts of hot water from routine outlet use. We report that this system can reliably induce resistance to heat shock in adapted lineages through underlying mutations that cause differences both in transcriptional regulation and heat-shock protein activity. In our model, adaptation to heat shock resulted in no fitness deficits during axenic or infectious growth, supporting epidemiological studies that connect environmental isolates sourced from hot water systems to clinical isolates from exposed individuals.

## RESULTS

**Fig 1:**
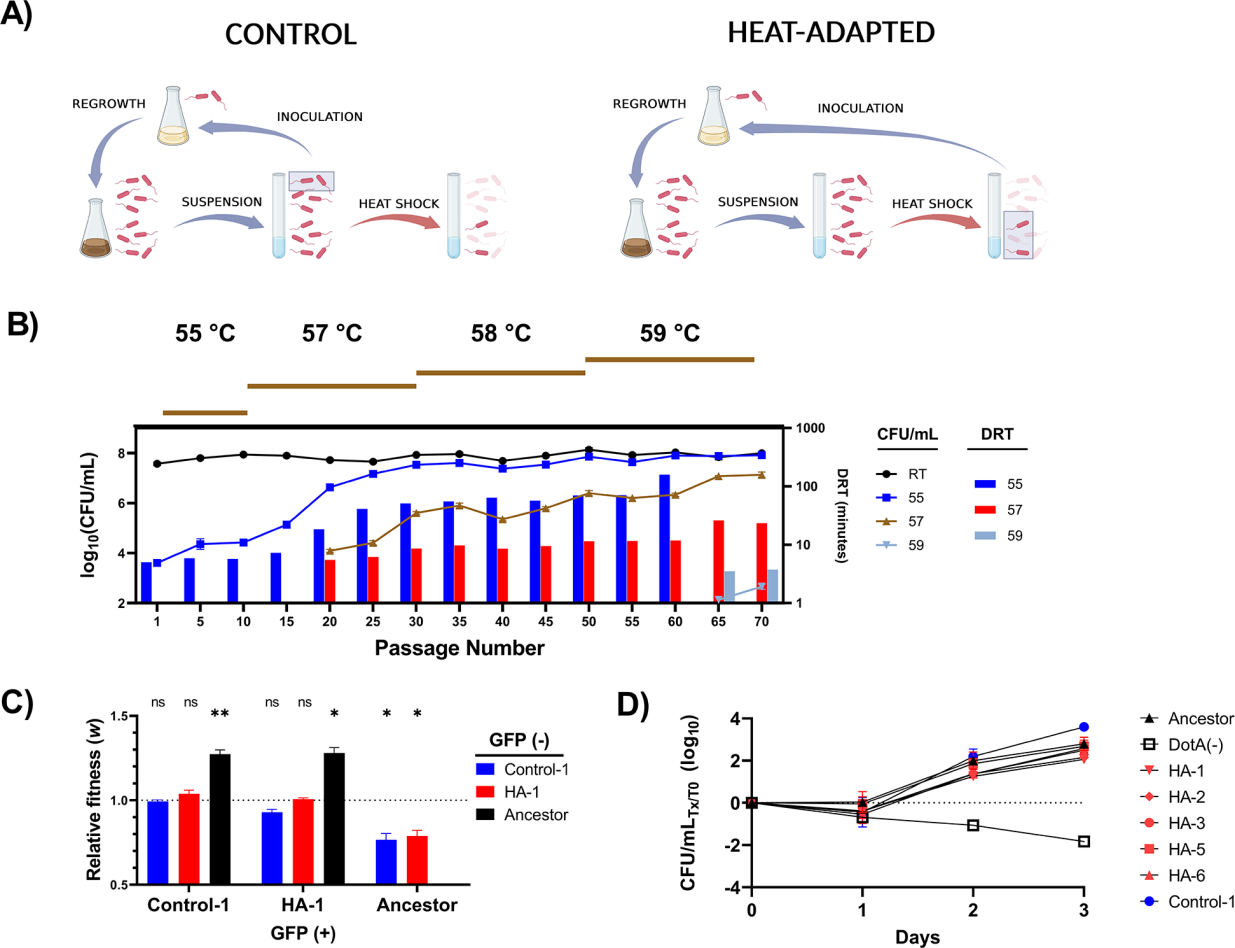
Adaptive laboratory evolution increased the resistance of *L. pneumophila* to heat shock. (A) Brief schematic showing the workflow of each passage in the evolutionary model for both the control and heat-adapted branches (created with BioRender). Each panel depicts 1 of 70 cycles, with highlighted rectangles showing the samples used to propagate the next passage. In brief, after collection from post-exponential growth in AYE and 24 h suspension in Fraquil, control lineages were propagated by subsampling the suspended population to re-inoculate AYE while heat-adapted lineages were propagated by re-inoculating AYE with the population surviving heat shock. (**B**) Survival of heat-adapted *L. pneumophila* lineages following 20 min of exposure to 55°C, 57°C, and 59°C heat shock for 20 min (lines, left axis). Decimal reduction times (DRT) were calculated for each temperature as the amounts of time required for a 90% reduction in population count and are plotted for each tested passage (bars, right axis; omitted for passages 65 and 70 at 55°C because there was an insignificant level of cell death). Survival was measured independently (with three technical replicates) for each of six heat-adapted lineages at five passage intervals. Data show mean of all lineages with error bars showing ±SD, *n* = 6. (**C**) Direct competition between green-fluorescent isolates and non-fluorescent isolates growing in AYE. Significance calculations show one-sample *t*-test against 1.0 (neutral relative fitness), *n* = 3. (**D**) Infection and replication within *V. vermiformis* in modified PYNFH without FBS, *n* = 3. The avirulent DotA(−) strain is used as a negative control.

Studies tracking the population structure over time of *L. pneumophila* growing in water distribution systems have repeatedly confirmed that persistence and microevolution are more common than recontamination from the incoming water supply (14, 60). Even more troubling, repeated exposure to high temperatures through bursts of hot water demand or failed cycles of superheat-and-flush is liable to favor heat resistance in the surviving bacteria. To simulate this process and study it in a controlled environment, we designed an adaptive evolution system to simulate transient and sublethal exposure of *L. pneumophila* cells to high temperatures. Broadly, *L. pneumophila* has a life cycle which alternates between a replicative phase to support intracellular multiplication in protozoa and a transmissive phase in which the bacteria search for new host cells to infect (22). Laboratory analogs of these regimes are seen in exponential phase and post-exponential phase cells grown in axenic culture, with short-term starvation enhancing the expression of some virulence factors and increasing stress resistance (61). To control for the variability of municipal tap water, we designed our experiment to include a 24-h suspension in Fraquil, a defined medium which simulates North American freshwater and supports the long-term survival of *L. pneumophila* (62), of cells harvested from post-exponential phase cultures to parallel planktonic bacterial populations in a plumbing system. Based on pilot data and typical global public health recommendations (15), we exposed the study populations to 55°C by immersion in a circulating water bath for 15 min, as described in Materials and Methods. We maintained six replicate lineages of the wild-type Philadelphia-1 strain to determine the replicability of our evolutionary pathways under heat shock (Fig. 1A). In parallel, we also maintained six replicate control lineages to compensate for mutations that favor growth in laboratory conditions and isolate the changes related to heat shock (Fig. 1A). Bacterial population sizes were bottlenecked at each cycle by either selection for survivors of heat shock or by subsampling of control populations suspended in freshwater.

Over the 70 cycles of the experimental model, we raised the heat-shock temperature on an *ad hoc* basis to maintain a stable level of population loss in the heat-adapted lineages from 55°C at the beginning of the experiment to 59°C at its conclusion (Fig. 1B). The resulting six heat-adapted populations were named HA-1 through HA-6, and the six control lineages were named C-1 through C-6 (Table 1). Whole-genome sequencing revealed that HA-4 had been contaminated by HA-6 by the end of the experiment, so it was removed from final analysis. Regardless, all replicate heat-adapted lineages showed a robust increase in heat-shock resistance when challenged by heat shock for 20 min (Fig. 1B). The decimal reduction time required to reduce the initial populations by 90% increased from 303 s at 55°C at the outset to a minimum of 9,546 s by passage 60 (Fig. 1B). After 65 and 70 cycles of selection, there was no significant population drop even after a 30-min exposure. After 20 and 65 passages, respectively, populations could survive 57°C and 59°C for 20 min. In contrast, neither the control lineages nor the ancestral strain was able to tolerate 57°C for 20 min (data not shown).

**TABLE 1:** Bacterial strains and plasmids used in this study. Except where noted, all strains used were of *L. pneumophila*

Strain	Description	Source
Philadelphia-1	Philadelphia 1976	ATCC33152
KS79	JR32 ∆comR	(63)
HA1-6	Replicate lineages derived from Philadelphia-1 through 70 passages under heat-shock selection	This study
C1-6	Replicate lineages derived from Philadelphia-1 through 70 passages without heat-shock selection	This study
Δ*lidA*	KS79 *lidA*::Kn^pSF6^	This study
*dnaK*^M94I^	KS79 dnaK::dnaK^M94I^ − Cm^pMMB207c^	This study
*dnaK*^M94I, V373L^	KS79 dnaK::dnaK^M94I, V373I^ − Cm^pMMB207c^	This study
Δ*htpG*	KS79 *htpG*::Kn^pSF6^	(64)
*htpG*^G83E^	KS79 *htpG*::*htpG^HA-3^ + Kn ^pGEMT-easy^*	This study
*htpG*^Q148*^	KS79 *htpG*::*htpG^HA-5^ + Kn ^pGEMT-easy^*	This study
*htpG*^Δ1bp nt603^	KS79 *htpG*::*htpG^HA-6^ + Kn ^pGEMT-easy^*	This study
*E. coli* DH5α	supE44 ∆lacU169 (Φ80lacZ∆M15) hsdR17 recA1 endA1 gyrA96 thi-1 relA1	Invitrogen
**Plasmid**	**Description**	**Source**
pMMB207c	RSF1010 derivative, IncQ, lacIq, Cm^r^, Ptac, oriT, ∆mobA	(65)
pXDC39	pMMB207c ∆Ptac, ∆lacI, Cmr	Xavier Charpentier
pSF6	pGEMT-easy-rrnb	(66)
pXDC31	pMMB207C + *gfp*	(67)
p*htpG*^G83E^	pXDC39 + *htpG*^HA-3^	This study
p*htpG*^Q148*^	pXDC39 + *htpG*^HA-5^	This study
p*htpG*^Δ1bp nt603^	pXDC39 + *htpG*^HA-6^	This study
p*htpG*^WT^	pXDC39 + *htpG*^Philadelphia-1^	(64)

Despite their acquired ability to tolerate heat shock, the heat-adapted lineages did not lose the ability to grow axenically or in host cells. As there appeared to be no substantial inter-lineage differences in growth rate during the adaptive laboratory evolution experiment, we chose to co-culture representative lineages HA-1 and C-1 against each other and against the ancestral Philadelphia-1 strain in AYE to test for relative fitness. Plasmids pXDC31 and pMMB207c, which differ only in that pXDC31 has an IPTG-inducible *gfp* insert, were used to distinguish between strains. Both plasmids were electroporated into the Philadelphia-1 ancestor as well as Passage 70 populations of both HA-1 and C-1, and transformed isolates were used in the competition experiments. To control for possible fitness confounds introduced by the plasmid inserts, all pairs were tested with a reciprocal expression of *gfp* in both competitors (e.g., HA-1 pXDC31 was competed against Philadelphia-1 pMMB207C and HA-1 pMMB207C was competed against Philadelphia-1 pXDC31). Control competitions of HA-1 and C-1 with themselves showed a neutral relative fitness, as expected (*P* = 0.4123, 0.5233). HA-1 and C-1 also competed at neutral relative fitness against each other (*P* = 0.00547, 0.2113), indicating that both were equally well adapted to growth in AYE. In contrast, Philadelphia-1 was significantly less fit than either HA-1 (*P* = 0.0120, 0.0235) or C-1 (*P* = 0.0090, 0.0244), supporting our hypothesis that the populations would evolve better growth in AYE in tandem with heat resistance. To test whether this increased growth potential in AYE was replicated during host cell infection, we tested for 3-day bacterial yield after growth in the amoeba *V. vermiformis*. Consistent with a specific adaptation to growth in cell-free conditions, replication of the heat-adapted lineages did not significantly differ from those of Philadelphia-1 or the control lineage (Fig. 1D).

**Fig 2:**
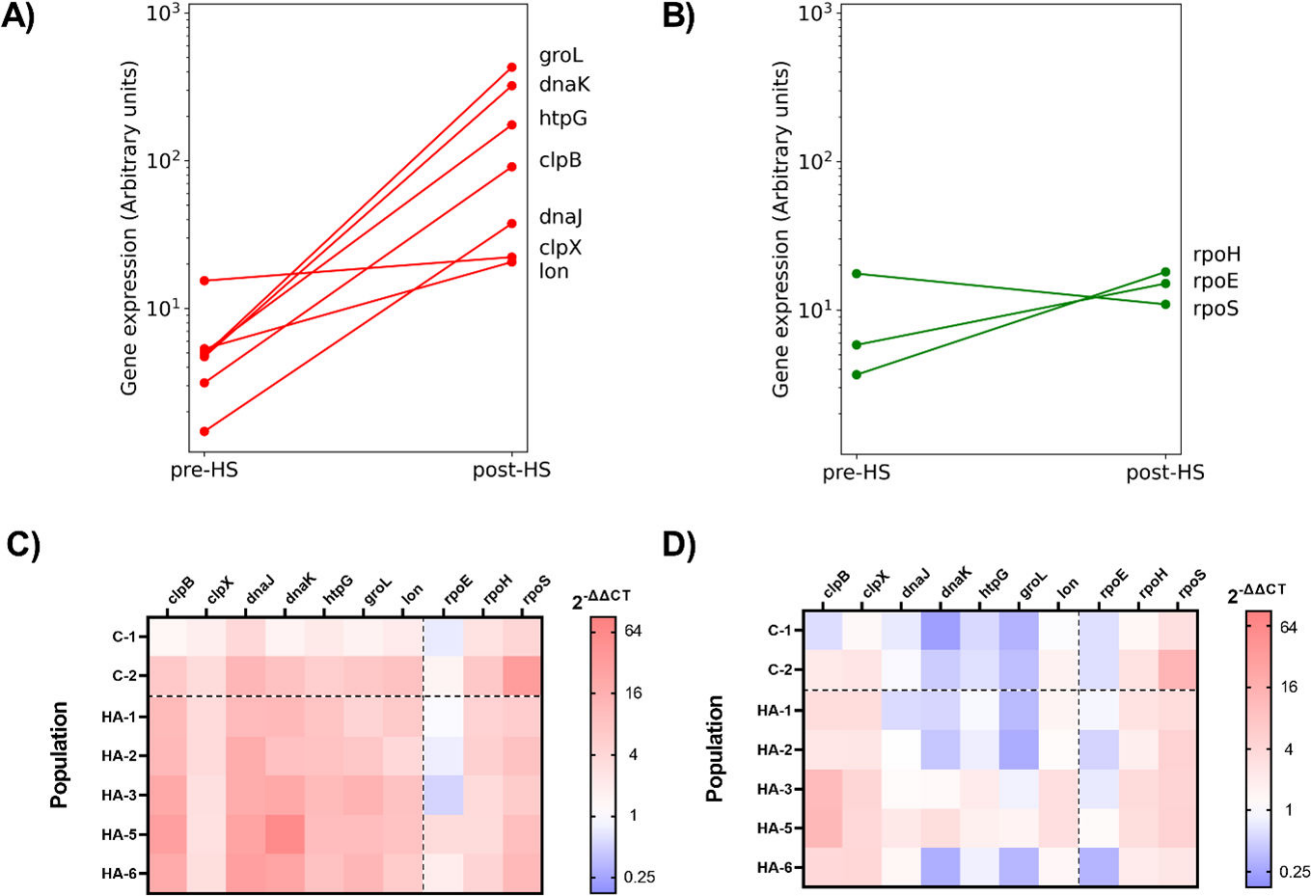
qPCR analysis of RNA expression levels in the ancestor Philadelphia-1 following 5 min of exposure to 55°C heat shock for heat-shock response genes (**A**) and sigma factors (**B**). Ct values were normalized using 16S rRNA levels and converted to arbitrary expression levels, *n* = 3. Relative difference in expression in RNA samples collected from control lineages (C-) and heat-adapted lineages (HA-) before heat shock (**C**) and after heat shock (**D**) compared to Philadelphia-1, *n* = 3.

Because the early bacterial heat-shock response relies largely on a re-direction of transcription by the *rpoH* sigma factor, we expected that there would be a difference in RNA expression profiles between our heat-adapted, control, and ancestral strains. To represent the regulators and effectors of the transcriptional heat-shock response, we measured differential expression of three sigma factors and seven heat-shock proteins. These were chosen to represent genes likely to be involved in the heat-shock response, as well as those mutated in multiple lineages under heat-shock selection. Upon heat shock, the ancestor Philadelphia-1 displays significant up-regulation in *clpB*, *dnaJ*, *dnaK*, *groL*, and *htpG* expression but not of *clpX* or *lon* by two-way ANOVA (Fig. 2A). Similarly, *rpoH* and *rpoE,* but not *rpoS,* were induced by heat shock, though it should be noted that there exists a significant degree of post-translational regulation of sigma factor activity (Fig. 2B). Compared to Philadelphia-1, the heat-adapted lineages (and to a lesser extent, the control lineages) over-express heat-shock protein mRNAs prior to heat-shock exposure (Fig. 2C). Comparing relative expression profiles after exposure to heat shock to the steady-state profile (Fig. 2D), this over-expression is greatly relieved suggesting a pre-adaptation to thermal stress in chronically heat-exposed populations with a less dramatic heat-shock response needed to survive transient exposure to high temperature.

**Fig 3:**
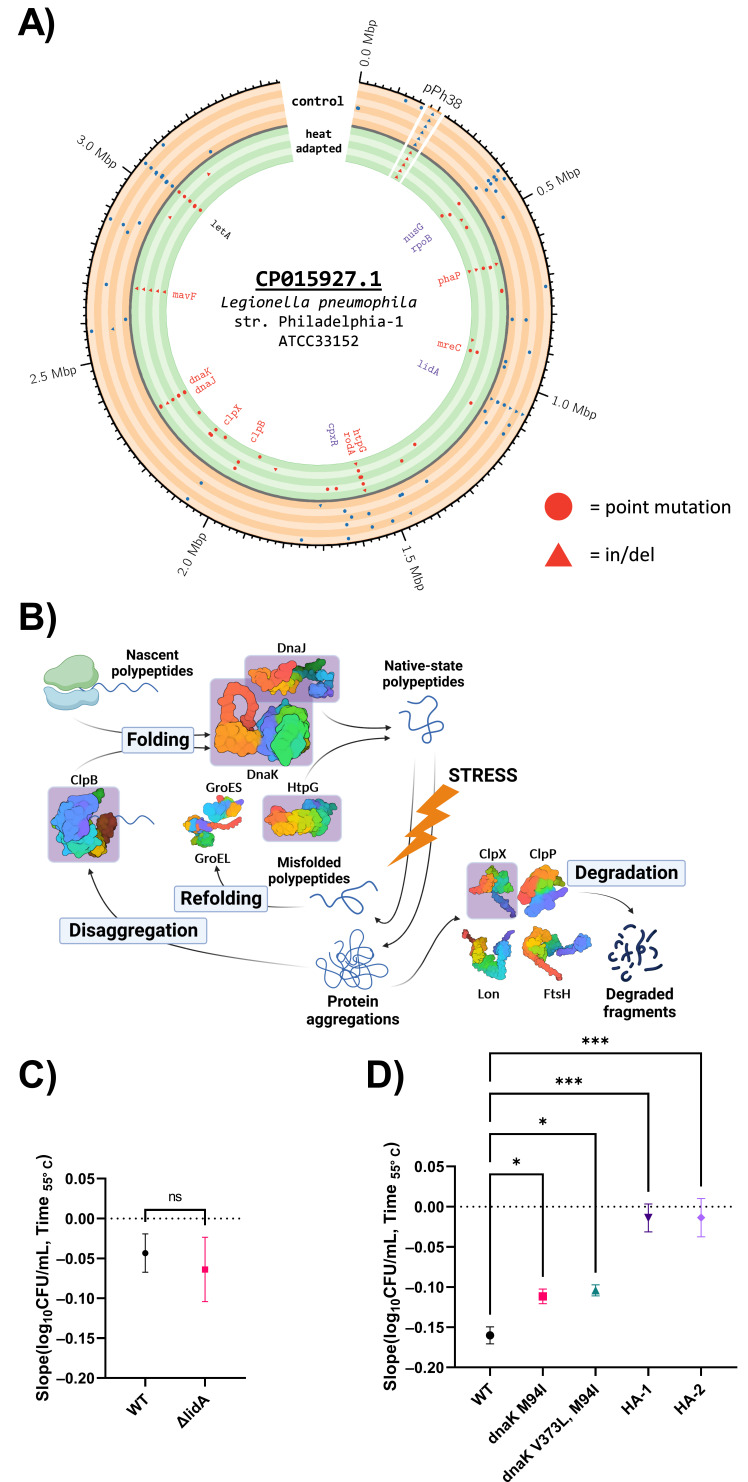
(**A**) Derived mutations identified with Breseq fixed in replicate control and heat-adapted lineages aligned to the genome of the ancestral strain Philadelphia-1 (68). The outermost concentric circle shows the single chromosome of *L. pneumophila*, as well as a reversibly integrated pPh38 element which was not retained in our lab strain. Each band shows the mutations observed across control lineage (orange) and heat-adapted (green), with circles representing point mutations and triangles representing insertions and deletions. Genes of interest in the interior are labeled by color with red genes mutated in multiple heat-adapted lineages and blue genes mutated in multiple control lineages. (**B**) Key participants in the bacterial heat-shock response include chaperones, proteases, and disaggregases, which act in a protein quality control network to direct proper protein function in nascent or damaged polypeptides. Proteins highlighted by a pink box acquired mutations in this adaptive laboratory evolution system. Multimeric proteins are depicted as monomeric structures for simplicity (created with BioRender). (**C**) Rates of population decline during exposure to 55°C in a water bath do not significantly differ between wild-type KS79 and a *lidA* deletion mutant, *n* = 3. (**D**) Rates of population decline of mutant strain carrying *dnaK* alleles observed in heat-adapted lineages, wild-type KS79 (WT), and heat-adapted population (HA-1 and HA-2) during exposure to 55°C, *n* = 6 (WT) or *n* = 3 (the remainder). (**C**) and (**D**) Point and error bars show mean ± SEM of the linear regression between log_10_ population values and time of exposure measured over 15 min (**C**) or 30 min (**D**). Statistical significance computed from slope and SEM using (**C**) Student’s *t*-test or (**D**) ANCOVA with Holm-Šídák correction for multiple comparisons; * = *P* < 0.05,*** = *P* < 0.0001 .

To identify the mutations acquired after 70 cycles of adaptive laboratory evolution, we used Breseq, a pipeline designed for the analysis of short read data from bacterial evolution experiments. To capture the population diversity, we isolated 10 clones from each heat-adapted lineage and 5 clones from each control lineage for whole-genome sequencing. As described in Materials and Methods, per-isolate analysis of the sequence reads showed gaps of missing coverage, so the reads were merged for each lineage, and analysis was performed to identify only mutations that were fixed in 100% of reads. Resequencing of our Philadelphia-1 strain showed that it differs from the published genome (CP015927.1) by the absence of a 38-kbp island pPh38 and by a missense mutation in the *letA* gene (Fig. 3A; Table 2). Genes mutated in replicate heat-adapted lineages included multiple heat-shock genes, including *htpG*, *clpB*, *clpX*, *dnaJ*, and *dnaK* (Fig. 3B). We also detected mutation in a phasin (*phaP*), an uncharacterized Icm/Dot effector (*mavF*), and genes associated with cell wall synthesis (*mreC* and *rodA*) (Fig. 3A; Table 3). A separate mutation profile was seen in the replicate control lineages, with multiple hits in *nusG*, *rpoB*, *cpxR*, and *lidA* (Fig. 3A; Table 4). Although each heat-adapted lineage displayed a unique mutational profile, overlaps in the identity of mutated genes show that plasticity in a small set of genes, including known heat-shock proteins, may form a core foundation for multiple evolutionary pathways toward heat resistance (Table S1).

**TABLE 2:** Resequencing of Philadelphia-1 to assess the ancestral state at the onset of the adaptive laboratory evolution model compared to the published genome CP015729.1

Gene	Name	Philadelphia-1	Gene ontology
	pPh38	Δ37,890 bp chr1: 173323	
Two-component system response regulator	LetA	L68R	Phosphorelay signal transduction system, regulation of DNA-templated transcription, DNA binding

**TABLE 3:** Selected mutations from heat-adapted lineages selected through heat-shock exposure after 70 passages^*a*^

Gene	Name	HA-1	HA-2	HA-3	HA-5	HA-6	Gene ontology
	pPh38	Δ37,890 bp chr1: 173323	Δ37,890 bp chr1: 173323	Δ37,890 bp chr1: 173323	Δ37,890 bp chr1: 173323	Δ37,890 bp chr1: 173323	
Two-component system response regulator	LetA	L68R	L68R	L68R	L68R	L68R	Phosphorelay signal transduction system, regulation of DNA-templated transcription, DNA binding
DNA-directed RNA polymerase subunit beta	RpoB	G138E			K924N		DNA-templated transcription, DNA binding, DNA-directed 5'−3' RNA polymerase activity, ribonucleoside binding
Phasin domain-containing protein	PhaP	Δ1bp nt39	Δ1bp nt39			+A 38	
Cell shape-determining protein	MreC	V266A	V266A				Regulation of cell shape
Chaperone protein	HtpG			G83E	Q148*	Δ1bp nt503	ATP binding, ATP hydrolysis activity, ATP-dependent protein folding chaperone, unfolded protein binding
Cell elongation protein	RodA		R112S	P332S			Cell division, cell wall organization, peptidoglycan biosynthetic process, regulation of cell shape, peptidoglycan glycosyltransferase activity
Chaperone protein	ClpB			A478V	A492T		Protein refolding, response to heat, ATP binding, ATP hydrolysis activity
ATP-dependent Clp protease ATP-binding subunit	ClpX	G202A	E203D				ATP binding, ATP hydrolysis activity, ATP-dependent protein folding chaperone, protein dimerization activity, unfolded protein binding
Heat-shock 40 kDa protein	DnaJ	F95Y	F95Y	P334S	+C 1100	R318T	DNA replication, protein folding, response to heat, ATP binding, heat-shock protein binding, unfolded protein binding
Heat-shock 70 kDa protein	DnaK	M94I	V373L, M94I			M94I	ATP binding, ATP-dependent protein folding chaperone, unfolded protein binding
Uncharacterized protein	MavF	Δ1bp nt368	Δ1bp nt368	Δ1bp nt368	Δ1bp nt368	+T 456	
NADH-quinone oxidoreductase	NuoG				G455D	E768K	ATP synthesis coupled electron transport, two iron, two sulfur cluster binding, four iron, four sulfur cluster binding, metal ion binding, NADH dehydrogenase (ubiquinone) activity, quinone binding

^*a*^ Genes were selected for inclusion based on their mutation in multiple replicate lineages. All mutations were fixed in the terminal populations—found in 100% of pooled reads aligned using Breseq from each lineages’ isolates. See Table S1 for full list of mutations.

**TABLE 4:** Selected mutations from control lineages naïve to heat-shock exposure after 70 passages^*a*^

Gene	Name	C-1	C-2	C-3	C-4	C-5	C-6	Gene ontology
	pPh38	Δ37,890 bp chr1: 173323	Δ37,890 bp chr1: 173323	Δ37,890 bp chr1: 173323	Δ37,890 bp chr1: 173323	Δ37,890 bp chr1: 173323	Δ37,890 bp chr1: 173323	
Two-component system response regulator	LetA	L68R	L68R	L68R	L68R	L68R	L68R	Phosphorelay signal transduction system, regulation of DNA-templated transcription, DNA binding
Transcription Termination/antitermination protein	NusG		R136I				V143D	DNA-templated transcription elongation, DNA-templated transcription termination, regulation of DNA-templated transcription elongation, transcription antitermination, transcription elongation-coupled chromatin remodeling
DNA-directed RNA polymerase subunit beta	RpoB				G759E	N1350T		DNA-templated transcription, DNA binding, DNA-directed 5'−3' RNA polymerase activity, ribonucleoside binding
30S ribosomal protein S10	RpsJ				Q36K	S102N		Translation, structural constituent of ribosome, tRNA binding
LidA	LidA	Δ1bp nt1566	Δ1bp nt1122	E508*	Δ1bp nt1566	Δ1bp nt1122	Δ1bp nt1566	
Cell elongation protein	RodA			A128T				Cell division, cell wall organization, peptidoglycan biosynthetic process, regulation of cell shape, peptidoglycan glycosyltransferase activity

^*a*^ Genes were selected for inclusion based on their mutation in multiple replicate lineages. All mutations were fixed in the terminal populations—found in 100% of pooled reads aligned using Breseq from each lineages’ isolates. See Table S1 for full list of mutations.

As we were interested in modeling microevolution in genuine water systems, we compared the mutations that our heat-adapted lineages acquired against 297 deposited genomes from the NCBI Genome database of *L. pneumophila* genomes and scaffolds, as well as from known published genomes from hot water distribution system (55 – 57; Table S2). With two exceptions, all mutations were unique while their surrounding residues or nucleotides were conserved across the remaining genomes (Table S3). ClpX^E203D^ was observed in both sample HA-2 and in isolate NY11, a clinical sample that was genetically linked to the environmental isolate NY12 from potable water that lacked this mutation. DnaK^M94I^ was identified in HA-1, HA-2, and HA-6 as well as in 64 other isolates (Table S2). Metadata associated with the sequence assemblies did not support an enrichment of this mutation in isolates with an environmental experience of heat-shock exposure (69, 70). Instead, the distribution of DnaK^M94I^ was contingent on the isolates’ clustering in the subpopulation structure of the species (71). Environmental isolates of the *Legionella pneumophila* subsp. *pneumophila* clade shared the ancestral allele at this locus with Philadelphia-1. In contrast, all assemblies (71 – 74) representing the *pascullei*, *raphaeli, fraseri*, and D-7708 (71) subspecies cluster (comprising 64/302 or 21.2% of analyzed assemblies) contained the derived DnaK^M94I^ allele. The uneven distribution of *Legionella* subspecies has been proposed to reflect an underlying occupation of separate ecological niches (71), so we chose to conduct follow-up experiments on the *dnaK* mutations observed in our heat-adapted lineages.

As an experimental test of their effects on heat-shock survival, we tested mutations in two proteins from either branch of the system. LidA received three separate mutations causing frameshifts or pre-mature stops in control lineages but none in heat-adapted lineages (Table 4), suggesting that loss of LidA function might be detrimental to survive heat shock. This is surprising as LidA is an Icm/Dot-translocated Rab GTPase-binding effector protein whose role in infection seems to largely involve disruption of the host trafficking system (75, 76). We constructed a *lidA* deletion mutant in the KS79 strain, a descendant of Philadelphia-1 used here as wild type for follow-up due to its genetic tractability. We observed no difference in the rates of population loss between the wild-type strain and the *ΔlidA* mutant after 15 min of exposure (Fig. 3C), an expected null phenotype for a gene with no clear connection to heat-shock survival and which was not observed in any heat-adapted lineages. *DnaK*, with mutations at two loci in three heat-adapted lineages, represented mutations acquired in the heat-adapted lineages, especially in light of the phylogenetic results described above. Unable to construct a full deletion mutant *dnaK* in our prior work (64), we instead constructed mutants in the KS79 background with the wild-type allele replaced by either of the two alleles acquired during the experiment—DnaK^M94I^ in HA-1 and HA-6 and DnaK^M94I, V373L^ in HA-2. There was a significant decrease in the speed of population loss at 55°C in the DnaK^M94I^ and DnaK^M94I, V373L^ mutants compared to the wild-type ancestor (Fig. 3D). Although adaptations in this single heat-shock protein were partially explanatory for the heat resistance of the final populations, comparisons with the fully adapted populations clearly show that these alleles can only contribute a portion of the fully evolved heat resistance phenotype.

**Fig 4:**
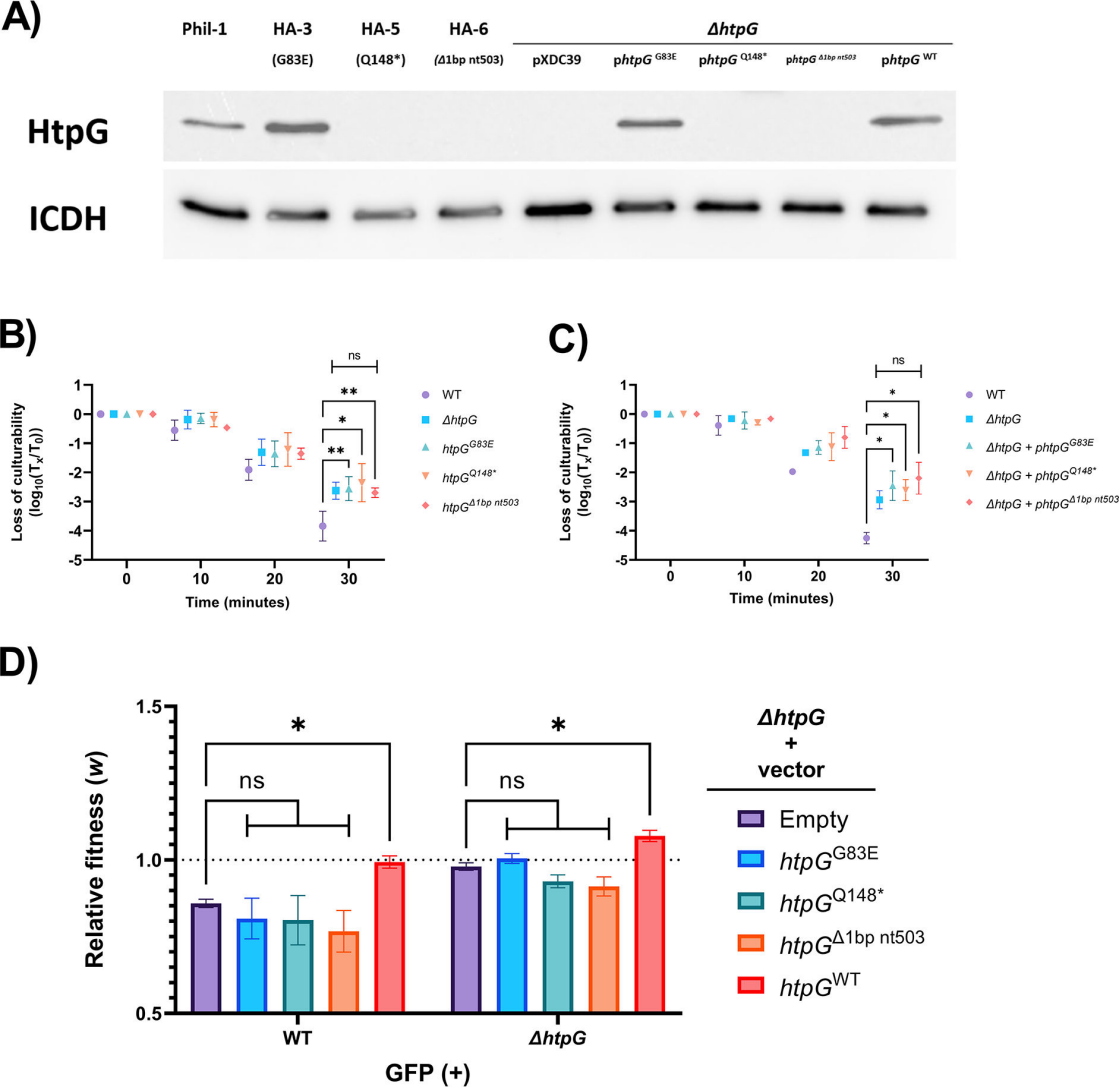
(**A**) Anti-*htpG* Western blots visualizing the expression of HtpG in lineages HA-3, 5, and 6 and in constructed merodiploid mutants (representative image). (**B**) Survival relative to initial population size of knock-in exchange mutants expressing derived alleles of *htpG* in place of the wild-type allele over a 30-min challenge of heat shock at 55°C, *n* = 3. (**C**) Survival relative to initial population size of an *htpG* deletion mutant heterologously expressing different alleles of *htpG* found in treated lineages over a 30-min challenge of heat shock at 55°C, *n* = 3. (**D**) Relative fitness of the *htpG* deletion mutant complemented with an empty plasmid, derived, or ancestral *htpG* alleles against the wild-type and deletion mutant strains over a 24-h incubation in AYE, *n* = 3. (**B and C**) Data shown represent mean population surviving relative to population size prior to heat shock (i.e., time 0) ± SD. (**D**) Data show mean relative fitness ± SD. Statistical significance calculated with two-way ANOVA in Prism 5.3.1 with Holm-Šídák correction for multiple comparisons; * = *P* < 0.005, ** = *P* < 0.01.

The *L. pneumophila* homolog of *htpG* (bacterial 90 kDa heat-shock protein) acquired mutations in three of the heat-adapted lineages including a nonsense mutation in HA-5 (Q148*) and a frameshift mutation in HA-6 (del1bp 503). Sequencing of HA-4 after 30 passages (before it had been contaminated) also showed a third distinct loss-of-function mutation (del1bp 694) (Table S4). Though surprising for a known heat-shock protein, this was consistent with our prior finding (64) that a deletion mutant lacking *htpG* showed increased survival under heat stress. Western blotting confirmed that the typical HtpG protein was not produced in HA-5 or HA-6, while the missense mutation (G83E) in HA-3 did not affect antibody binding (Fig. 4A). The *htpG* alleles found in HA-3, HA-5, and HA6 were mobilized by allelic exchange in tandem with a kanamycin-selectable marker into the chromosome of the *L. pneumophila* strain KS79. Phenotypically, the mutant alleles of *htpG* raised heat-shock resistance in *L. pneumophila* to a similar magnitude as the previously constructed full deletion mutant (Fig. 4B). Reciprocally, none of the mutant alleles were able to complement the raised heat resistance of the deletion mutant (Fig. 4C).

To test whether *htpG* mutation was favored only due to its role in heat shock, we constructed a competition assay to compare the growth of KS79 and of the deletion mutant against the mutant harboring the different alleles of *htpG* (Fig. 4D). The wild-type KS79 under-competed against any strain containing a blank plasmid or plasmid carrying any derived allele of *htpG*. Conversely, it was neutrally fit against the *htpG* mutant complemented with a wild-type allele of *htpG*. KS79 and the deletion mutant both grew significantly better in the presence of competitors harboring the ancestral allele of *htpG* compared to competitors harboring a blank plasmid, suggesting that HtpG, in addition to conferring reduced heat-shock resistance, may also reduce growth potential in AYE.

## DISCUSSION

### *L. pneumophila* rapidly adapts to repeated exposure to heat shock

As an opportunistic colonizer of plumbing infrastructure, *L. pneumophila* is commonly recognized as a major hazard under the purview of hospital facilities managers (10). To model its long-term residence in hot water systems, our system of laboratory evolution was able to robustly raise the heat tolerance levels of wild-type *L. pneumophila*. The lineages’ survival of 55°C—a commonly recommended circulation temperature (77, 78)—highlights the scale of their adaptation. Prior to their passage through the adaptive laboratory evolution model, the ancestral strain could survive only 5 min of exposure to this temperature before suffering a population reduction of 90% (Fig. 1B). After 65 rounds of selection, the heat-adapted lineages could sustain exposure to the same temperature for at least 30 min without showing significant population loss. The final selection condition—15 min at 59°C—was much more intense than what the ancestral strain could survive and indeed our control lineages, which were also tested for heat-shock survival, consistently failed to survive temperatures of 57°C and higher.

A major concern underlying our experiment was that *L. pneumophila*, accustomed to replication inside eukaryotes, might adapt to favor growth in liquid media and lose the ability to replicate inside host cells. To simplify the scope of our experiment, it was designed to model the exposure of planktonic *Legionella* cells to heat stress within a pasteurized plumbing system and did not include stages of intracellular replication or biofilm-associated growth. To account for their repeated axenic culture, we included replicate control lineages to anticipate the changes seen under this selection pressure. Testing populations from both branches of the evolution experiment, we found that both branches of the experiment retained their ancestral ability to replicate inside *V. vermiformis* (Fig. 1D). On the other hand, passage in axenic culture significantly elevated the relative fitness of both the control and heat-adapted lineages in AYE culture compared to Philadelphia-1 (Fig. 1C). Despite relaxed constraints during the selection of the control lineage, the heat-adapted lineage had an equal ability to grow in AYE or inside host amoeba without needing to trade-off the ability to tolerate heat shock.

### Tandem adaptation of transcription and translation during adaptation to axenic culture

Consistent with their separate evolutionary pressures, the control and heat-adapted lineages adopted divergent evolutionary patterns. RpoB, the beta-subunit of the RNA polymerase (RNAP) complex, is commonly mutated in bacterial evolution studies under selection for various phenotypes including survival of acid stress, ionizing radiation, and atmospheric pollution (54, 56, 79). Four different missense mutations in *rpoB* were independently fixed in two control lineages (G759E and N1350T) and in two heat-adapted lineages (G138E and K924N). As the only gene receiving multiple mutations in both the heat-adapted and control lineages, these mutations are more likely to be a basic response of *L. pneumophila* under our axenic passage conditions than a specific adaptation to heat-shock selection. From similar evolutionary experiments in *E. coli,* mutations in *rpoB* are known to cause broad changes to global transcription (80) and can optimize replication rates during exponential growth (81, 82). In these experiments, the same mutations in *rpoB* that contribute to faster exponential growth rates also reduce the ability of these strains to tolerate acid shock and antibiotic exposure (82). This may explain the distinction between the *rpoB* mutations found in the heat-adapted and control lineages as heat shock induces a hostile environmental change as well.

### Connections between virulence and heat-shock survival

Prior work in our lab has identified the two-component system response regulator *cpxR* as necessary for heat-shock tolerance in *L. pneumophila* (49). C*pxR* was mutated in three lineages in the control arm but in none of the heat-adapted lineages. Of the three distinct mutations, two converted aspartic acid to asparagine (D190N and D194N) in the OmpR/PhoB-type DNA binding domain (83, 84). Involved in controlling the expression of Type II and Type IV secretion system genes, the *cpxRA* system is implicated in the control of virulence during the switch between exponential and post-exponential phases (85, 86). As *cpxR* was not mutated in any heat-adapted lineage, there could be an underlying inelasticity that favors its mutation in control lineages but not in heat-adapted lineages.

Similarly, the *lidA* effector gene was mutated singly or doubly in each of the six control lineages but in none of the heat-adapted lineages. Each control lineage acquired either one of two frameshift mutations (single base deletions at nucleotides 1,122 or 1,566) or a premature stop (E508*). The role that these mutations in *lidA* play in this evolutionary system is obscure considering that (i) adaptive laboratory evolution was carried out without passage through host cells, (ii) effector proteins are thought to be highly redundant and the repeated mutation of *lidA* is *a priori* unexplained, (iii) *lidA* mutation seems to have been highly selected for in control lineages but was not observed during heat adaptation, and (iv) deletion of lidA does not reduce heat-shock survival. The study which characterized *lidA* found that its absence caused the Icm/Dot complex to become toxic to the bacterium, which was attributed to a possible toxic increase of solute permeability (87). An increase of permeability following the loss of LidA may support increased nutrient uptake in AYE, though the factors favoring complete saturation of *lidA* mutations across all the control lineages and none of the heat-adapted ones remain unclear.

In contrast, the effector *mavF* acquired frameshift indels in multiple heat-adapted lineages. Each of the five heat-adapted lineages acquired one of two distinct null mutations in *mavF*—a single base pair deletion of nucleotide 368 or a single base pair insertion at position 456. The biological mechanism favoring this mutation is difficult to deduce as the protein is translocated through the Icm/Dot system and, therefore, has no *prima facie* relevance to heat-shock survival in the absence of host cells (88). Across a nucleotide alignment of clinical and environmental isolates, this locus was markedly variable (Table S3), which could be a reflection of its relative plasticity as a translocated and potentially redundant effector (89).

### Central role of the DnaJ/DnaK/ClpB disaggregase complex in heat-shock adaptation

Adaptation to heat stress, as expected, took multiple unique but overlapping pathways. Canonical heat-shock factors were common targets of mutation and included *htpG, clpB*, *clpX*, *dnaJ*, and *dnaK*. These are, various, chaperone and co-chaperone proteins that act on misfolded and aggregated proteins to resolve the effects of protein damage (90). The *bona fide* HSP40 co-chaperone *dnaJ* was mutated in all five heat-adapted lineages, while neither of the HSP40 homologs *cbpA* or *djlA* (91 – 93) received any mutations. Four distinct mutations were acquired in *dnaJ*, including three missense mutations and a single nucleotide insertion. Three mutations—P334S, R318T, and an insertion at nucleotide 1,100 (codon 337)—are in the C-terminal end of the polypeptide. This region has been linked to the binding between DnaJ and DksA, a regulator of RNA polymerase and the stringent response which influences heat-shock resistance in *L. pneumophila* (64, 94 – 96). Autodimerization and chaperone functions in DnaJ also involve this C-terminal region (97). Two lineages derived the same mutation—F95Y—in a linker region connecting the J-domain and central zinc-binding regions of DnaJ. This phenylalanine position is conserved across bacterial and eukaryotic homologs of DnaJ as a residue in the first of three DIF (DVF in *Legionella*) motifs (98). Experimental substitution of this residue in *E. coli* causes a growth defect at low temperatures but has no effect on high-temperature growth or on motility. The integrity of this glycine-phenylalanine-rich region is thought to be involved in proper DnaK/DnaJ ATP cycling and substrate release (98). This mutation in DnaJ may contribute to the constitutively high expression of heat-shock genes seen in our heat-adapted lineages by causing an accumulation of DnaK-substrate complexes and causing a de-repression of steady state *rpoH* mRNA levels (99).

DnaK, a bacterial HSP70 and collaborator with DnaJ in protein refolding, acquired the same mutation in three lineages—M94I. This locus was polymorphic in our panel of tested genomes, coinciding with a broader phylogenetic divergence between subspecies *pneumophila* and other clades. Conceivably, this could be an adaptation contributing to a speculated difference in preferred ecological niches (71) between the subsp. *fraseri*, *pascullei*, D7708, and *raphaeli* isolates which contain this mutation and the subsp. *pneumophila* which do not. F95Y mutations in DnaJ found in HA-1 and HA-2 were coincident with this DnaK allele, with HA-2 also possessing a second V373L missense mutation in DnaK. These mutations both alter residues exposed on the AlphaFold-predicted surface of the DnaK protein (100, 101), suggesting that their effects lie in modification of the substrate binding or co-chaperone dynamics of the heat-shock response network. Testing the mutations in *dnaK* in isolation on a wild-type background (Fig. 3D) showed that both protein-level changes were beneficial alone in promoting heat-shock survival but could not raise the heat resistance of the strain to match the robust heat tolerance of the fully evolved lineage.

The DnaK/DnaJ chaperone-co-chaperone system also interacts with ClpB, or HSP100, a AAA +ATPase with homolog found in all three domains of life (102 – 104). In various species of bacteria, ClpB assists in the tolerance of high temperatures, ethanol damage, and oxidative stress by acting as a disaggregase of tangled polypeptides (102). Its proper function depends on the assembly of an active hexamer from ClpB monomers, forming a central channel that the misfolded substrate is threaded through. Interaction with DnaK, as well as hexameric assembly of the protein complex, depends on the M-domain of ClpB (105, 106). This section of the larger AAA+ domain includes four surface-exposed α-helices and spans the two mutations observed in ClpB (A478V and A492T). The presumed effect of these mutations is to influence protein–protein interactions either during self-hexamerization or when interfacing with DnaK (107, 108).

### Refinement of the heat-shock response by protein degradation

A second hexameric AAA+ ATPase, ClpX, also showed signs of adaptation with mutations in two lineages targeting adjacent residues: G202A and E203D. Unlike ClpB, ClpX favors proteolysis over refoldase activity, acting in concert with the peptidase ClpP (109). The two mutated residues are both in the AAA+ domain of ClpX and may allow for a refinement of the proteolytic capacity of *L. pneumophila* that remediates the enrichment of unfolded and aggregated protein precipitated by high-temperature exposure.

As we had previously observed (64), the 90-kDa heat-shock protein HtpG has an unlikely role in the heat resistance of *L. pneumophila*. Its up-regulation after heat shock, role in high-temperature growth in other species, and its chaperone function all strongly support its identity as a heat-shock protein (110 – 112), but the deletion of *htpG* from a wild-type background increases the ability of *L. pneumophila* to survive exposure to 55°C for 15 min. This is further corroborated by the observation of three different mutations observed in three of the heat-adapted lineages. Two of these mutations—Q148* in HA-5 and a single base pair deletion at position 503 in HA-6—abrogate the structure of the chaperone, leading to the loss of recognition by an anti-HtpG antibody. These three alleles provide the same resistance to heat shock as the full deletion of *htpG* and do not complement the deletion. We hypothesized that these presumed loss of function mutations may give a growth advantage between heat-shock bottlenecks. We tested this in a relative fitness experiment measuring the growth of a deletion mutant heterologously expressing the wild-type and derived alleles of *htpG*. Strains lacking *htpG* were more relatively fit than one expressing wild-type *htpG*. This was also true for all three derived alleles of *htpG*; the loss of HtpG function during the experiment could, therefore, have promoted both increased growth in AYE and survival under heat shock. As *htpG* mutations were only seen in our heat-adapted lineages, it seems likely that the increase in heat resistance is the more significant factor favoring their selection.

### Future avenues

With hospitals recognizing the dangers that *L. pneumophila* introduces when it contaminates their plumbing, it is important to understand its life in these systems before it causes nosocomial infection. Heat-based control measures are common, but *L. pneumophila* is often able to survive both routine hot water temperature regimes and remedial superheat-and-flush interventions (29, 113). Importantly, due to the severe consequences of *L. pneumophila* infection, a combination of disinfectants can also be deployed to combat contamination in plumbing systems. Although the dynamics of evolution under joint exposures to heat shock and other drinking water disinfectants were not in the scope of this study, the speed that our heat-adapted lineages were able to develop substantial heat resistance highlights the crucial importance of avoiding repeated cycles of futile and incomplete pasteurization. To increase the chances of full and effective clearance of *Legionella*, operators should at minimum meet or exceed the guidelines of hot water system temperatures and consider designing or retrofitting systems to permit additional treatments, such as copper–silver ionization, chlorination or chloramination, or UV light disinfection (11, 114). Our adaptive laboratory evolution model of *L. pneumophila* microevolution under selective pressure from transient heat shock showed several possible evolutionary pathways to acquire heat resistance and replicates *in situ* studies that find increased heat resistance in isolates taken after failed heat treatment (29). These mutations were concentrated both in well-known chaperones and proteases (e.g., *dnaJ*, *dnaK*, *clpX*, etc.), as well as in unexpected proteins with unrelated known functions (e.g., *phaP*, *mavF*). *L. pneumophila* lineages in this system evolved in isolation from the broader water system microbiome, but the horizontal transfer of resistance mechanisms is known as a broadly conserved phenomenon in multi-species bacterial systems and has previously been shown between different genotypes of *L. pneumophila* (115, 116). This model helps inform our understanding of the forces operating on *L. pneumophila* in hot water plumbing and provides a platform for future work to directly confirm the biological significance of the genetic changes that we have observed in this study.

## MATERIALS AND METHODS

### Routine culture methods

*L. pneumophila* strains (Table 1) were routinely grown from frozen stocks stored at −80°C on CYE (ACES-buffered charcoal yeast extract) agar plates (Sigma-Aldrich) adjusted to pH 6.90 with KOH and supplemented with 0.25 g/L L-cysteine and 0.4 g/L ferric pyrophosphate and incubated at 37°C for 72 h. Single colonies were subcultured into AYE broth (CYE without charcoal or agar) and grown shaking at 37°C overnight. To simulate residence in tap water, Fraquil, a defined low-nutrient medium (117) that simulates freshwater was used to suspend *L. pneumophila. E. coli* strain DH5α was grown in LB agar or broth, as indicated, at 37°C overnight. Where required, media were supplemented with 5 mg/L (*L. pneumophila*) or 25 mg/L (*E. coli*) chloramphenicol, 25 mg/L kanamycin sulfate, and/or 0.1 mM isopropyl β-d-1-thiogalactopyranoside (IPTG). The strains used in this study are listed in Table 1. *Vermamoeba vermiformis* was maintained in modified PYNFH (ATCC media 1034) at room temperature (20°C–28°C) and passaged once per week (49).

### Adaptive laboratory evolution procedure

The adaptive laboratory evolution experiment was designed with two arms deriving from one common ancestral genotype. *L. pneumophila* str. Philadelphia-1 (ATCC 33152) was obtained from the American Type Culture Collection; it is a clinical isolate collected from the 1976 outbreak of Legionnaires’ Disease in Philadelphia. Long-term laboratory culture of *L. pneumophila* is known to lead to the accumulation of mutations that are thought to adapt it to the novelties of cell-free growth that contrast with its typical dependence on infectious growth (68, 118). To account for this, we included both an experimental arm which was exposed to heat stress and a control arm which was not exposed to heat stress (Fig. 1A) to compensate for these effects.

To begin the experiment, a single culture of Philadelphia-1 was divided into 12 independent lineages to assess the parallelism between adaptive paths, divided into six evolved lineages that were exposed to heat shock and six control lineages that were not. Each population was grown in AYE overnight to stationary phase. Bacteria were triply washed in Fraquil, suspended at OD_600_ 0.1 (approximately 10^8^ CFU/mL), and incubated for 24 h at room temperature, as in reference (62). For each suspension, 1 mL was dispensed into a 13-mL polypropylene culture tube (Sarstedt). Tubes of the treated lineage were immersed for 15 min under ambient light in a pre-heated water bath set to the challenge temperature (55°C–59°C). Samples were withdrawn from the water bath and cooled passively to room temperature. Tubes of the control lineages were left at room temperature for the same length of time under ambient light as well. To synchronize growth between the experimental conditions and to equalize the effects of population bottlenecking on genetic drift, the size of the inoculum used to propagate each passage was approximately equalized between the two branches. Control lineages were propagated by inoculating 1.5 mL AYE with 15 µL (approximately 1.5 × 10^6^ CFU) of the untreated suspension to mimic the population bottleneck created by heat shock. Evolved lineages were propagated by inoculating 750 µL of 2× concentrated AYE with 750 µL of the heat-treated suspension. Cultures were grown overnight to stationary phase, as before, and a 100-µL aliquot was stored at −80°C in AYE + 15% glycerol to maintain a frozen archive of the full population at each passage.

The experiment was continued for 70 passages with the temperature of heat stress increased on an *ad hoc* basis to maintain the strength of selective pressure. Based on preliminary experiments, the experiment was begun with a 15-min exposure to 55°C. After 10 passages under selection, this was increased to 57°C. This was further increased to 58°C after 30 passages and finally to 59°C after 50 passages.

### Heat-shock survival measurements

Following the conclusion of the experiment, heat-adapted populations were resuscitated from frozen stocks, grown on CYE, and suspended in Fraquil as above. All lineages were tested at five passage intervals for heat-shock survival after 20 min of exposure to 55°C, 57°C, and 59°C in 60 µL volumes simultaneously in a Veriti 96-well thermocycler and actively cooled to 20°C, with control samples held at room temperature. CFU counts were determined by serial dilution and plating in 10 µL aliquots on CYE plates.

A similar strategy was used to determine the heat-shock tolerance of mutants expressing the various derived alleles of *htpG*. Samples were prepared and aliquoted as above and exposed to 55°C for 0, 10, 20, and 30 min in a Veriti 96-well thermocycler and actively cooled to 20°C. CFU counts were conducted as above and normalized to the CFU count of the 0-min exposure sample.

### Isolate collection and sequencing strategy

Single colonies were isolated from the populations generated after 70 passages and stored at −80°C. Ten isolates were collected from each heat-adapted lineage, and 5 isolates were collected from each control lineage. In addition, we resequenced our lab stock of Philadelphia-1 to identify any mutations that were fixed in the ancestral population at the outset of the experiment. DNA was collected from these isolates using the Wizard Genomic DNA Purification Kit (Promega) and submitted to the McGill Genome Center for library preparation and sequencing using a 600-cycle MiSeq v3 Reagent Kit.

Fastp (119) with default settings was used to both clean and perform quality control on paired-end reads. Read alignment and variant calling were conducted using Breseq 0.35.4 (120), bowtie2 2.3.5.1 (121, 122), and R 4.0.3 (123) with the NCBI-deposited sequence (CP015927.1) of the ancestral Philadelphia-1 ATCC 33152 strain as reference genome. Because the per-isolate read coverage was low (mean = 14.37, SD = 2.37), there were stochastically insufficient numbers of aligned reads to call allele states at every mutated locus in each of the sequenced isolates. To increase the depth of coverage, we analyzed pooled per-lineage sequence data by combining the reads together of all 10 sampled isolates for each heat-adapted lineage or all 5 sampled isolates for each control lineage to reconstruct the allele frequencies in the final populations. We re-analyzed these pooled reads using Breseq with the same parameters to generate our final list of called variants by filtering only for variants discovered at 100% (i.e., fixed in the passage 70 populations).

To characterize these mutations against the diversity of the *L. pneumophila* species, we compared the gene sequences derived from repeated selection against heat-shock survival against a list of *L. pneumophila* fully assembled genomes and scaffolds (297 total; Table S2) including cooling tower strains, potable water-derived strains, and clinical isolates. Ancestral and mutated coding regions were identified across the panel of genomes using BLAST 2.11.0 locally and aligned using Clustal Omega 1.2.4 (124). Position-count matrices were computed for: (i) 11 amino acids centered on non-synonymously mutated residues or (ii) 31 nucleotides centered on all other mutations (Table S3).

### qPCR protocol and analysis

RNA was collected from 1 mL samples of *L. pneumophila* suspended at an OD_600_ of 1, either heat shocked at 55°C for 15 min or held at room temperature for the same duration using TRIzol (ThermoFisher Scientific), as previously described. RNA samples were treated with DNase I and DNAse inactivation reagent (ThermoFisher Scientific) before quantification and storage in nuclease-free water (ThermoFisher Scientific) at −20°C until required. Protoscript II (New England Biolabs) was used to reverse transcribe RNA into cDNA as template for qPCR analysis using the manufacturer’s protocol. No-RT control reactions were produced in the same way with nuclease-free water replacing Protoscript II.

All qPCR experiments were performed in an Applied Biosystems 7500 Fast Real-Time PCR machine using iTaq Universal SYBR-green supermix (BioRad). Efficiencies for qPCR primers (Table 5) were calculated using a serial dilution of gDNA samples, and all were found to be acceptable (90.9%–96.8%) (125). 16S rRNA was used as a common housekeeping gene to normalize Ct values (64), and efficiency-adjusted ddCT (126) was used to compare RNA expression levels between bacteria exposed and naive to heat shock.

**TABLE 5:** All primers used during this study

Primer	Sequence 5'−3	Source
**Primers for qPCR**		
groL-qPCR-F	GAACATGGGCGCTCAAATGG	This study
groL-qPCR-R	GCAGCAACTGCTTTGTGACC	This study
lon-qPCR-F	GTTGCTTCAGCAGACATCGG	This study
lon-qPCR-R	GCTGGAATGCCGAAAGAAGC	This study
rpoH-qPCR-F	CCCATCTTGGGGTCAAAACG	This study
rpoH-qPCR-R	CGCGGTTATCTGGGGTATGG	This study
rpoE-qPCR-F	CCAGGTTGAAAACTGACTGCG	This study
rpoE-qPCR-R	CTGTTTGAGCGTTATCAGGGC	This study
rpoS-qPCR-F	ATGCGACCTGGTGGATTAGG	This study
rpoS-qPCR-R	TTTGCGTCAATTGCCTTGCC	This study
htpg-qPCR-F	TTACATACCAGCCCATGCCC	This study
htpg-qPCR-R	TCGGGGTAAAAATTGTGTCGC	This study
clpb-qPCR-F	CACTTGCAGATGCCCAATCC	This study
clpb-qPCR-R	AGAGGTCTGCAACTTCCACC	This study
clpx-qPCR-F	ATTAAGAATCCGCGCCAGGG	This study
clpx-qPCR-R	GAGGATGGTGTTGAGCTCGG	This study
dnaj-qPCR-F	TTTGTCCACGTGATTGACGC	This study
dnaj-qPCR-R	ATCCTTCAATGGGTGGAGGC	This study
dnak-qPCR-F	CGCAATAACAGAGTCACCGC	This study
dnak-qPCR-R	GCAACCAAAGATGCTGGTCG	This study
16s-qPCR-F	AGAGATGCATTAGTGCCTTCGGGA	(58)
16s-qPCR-R	ACTAAGGATAAGGGTTGCGCTCGT	(58)
**Primers for mutant construction**		
htpG-UF	CTTGCACATCTTGGAAAAGGAGG	(58)
htpG-UR	CAGTCTAGCTATCGCCATGTACTCCCCGTTAATAGTTGTGTTCG	(58)
htpG-KnF	CGAACACAACTATTAACGGGGAGTACATGGCGATAGCTAGACTG	(58)
htpG-KnR	GCTCATCCCGGATAATGACTTGTACCCAACTGATCTTCAGCATC	(58)
htpG-DF	GATGCTGAAGATCAGTTGGGTACAAGTCATTATCCGGGATGAGC	(58)
htpG-DR	CAATTATTGCCTTACTGCCTGGG	(58)
lidA-UF	CCTTACTTGAGGCTAGTTCGC	This study
lidA-UR	CAGTCTAGCTATCGCCATGTACATTTACTATCGTGCGATCTTGAG	This study
lidA-KnF	CTCAAGATCGCACGATAGTAAATGTACATGGCGATAGCTAGACTG	This study
lidA-KnR	GATTCTCAGATAATAATCCAGGACCCAACTGATCTTCAGCATC	This study
lidA-DF	GATGCTGAAGATCAGTTGGGTCCTGGATTATTATCTGAGAATC	This study
lidA-DR	CATTCAGTTGCTTATGGTCAGG	This study
dnaK-UF	GAATCCTTCTTCCGCCCTCT	This study
dnaK-UR	GCAGCGAATGCCGGGTAATAAATCGTCATGATGCACCCCAT	This study
dnaK-DF	CGTTCTTCGGGGCGAAAACTCAAGCTATCACCCTAAAAAAAGC	This study
dnaK-DR	ATGGACAATTACTGCAAGCCA	This study
dnaK-CmF	GCTTTTTTTAGGGTGATAGCTTGAGTTTTCGCCCCGAAGAACG	This study
dnaK-CmR	ATGGGGTGCATCATGACGATTTATTACCCGGCATTCGCTGC	This study
**Primers for complementation**		
KpnI-HtpG-F	TCATCAGGTACCTGAATCGAGAGGGTTTGAGTTGG	(58)
XbaI-HtpG-R	TCATCATCTAGAGGATAATGACTTGTTGTAATCCGGG	(58)

### Mutant construction

Mutants were constructed to perform functional follow-ups on the roles of *lidA* and *dnaK* mutations on a wild-type genetic background. A Δ*lidA* deletion mutant was constructed by allelic exchange and replaced with a kanamycin resistance cassette according to previously published protocol (64). Briefly, upstream fragment was amplified with primer lidA-UF and lidA-UR, the kanamycin cassette with lidA-KnF and lidA-KnR and the downstream fragment with lidA-DF and lidA-DR. The three fragments were then joined together by sewing PCR, and the resulting amplicon transformed into KS79 by natural competence. Previous efforts to construct a *dnaK* deletion mutant in *L. pneumophila* had failed (64), so a modified allelic exchange was used to construct the *dnaK*^M94I^ and *dnaK*^M94I, V373L^ mutants. Briefly, the desired sequences were amplified from isolates of HA-1 and HA-2, respectively, which contained the mutated loci in the upstream flanking region amplified by DnaK-UF and DnaK-UR. This fragment was sewed by PCR with a chloramphenicol cassette and a downstream fragment. The resulting amplicon was recombined into the wild-type KS79.

To complement HtpG, we constructed merodiploids by supplementing the various derived alleles of *htpG* on a plasmid. Plasmids were constructed on a pXDC39 backbone allowing *htpG* to be expressed under its native promoter. Using Phusion polymerase and primers kpni-htpg-F and xbai-htpg-R, we amplified the treated lineages T3 (*htpG*^G83E^), T5 (*htpG*^Q148*^), and T6 (*htpG*^Δ1bp 503nt^) alleles from the purified gDNA of single isolates from passage 70. These fragments, carrying the gene sequence and its upstream region, were ligated into pXDC39 to produce plasmids p*htpG*^G83E^, p*htpG*^Q148*^, and p*htpG*^Δ1bp 503nt^. These plasmids were electroporated into a previously constructed KS79-based *htpG* deletion mutant (64) to assay for relative fitness and to isolate the derived *htpG* alleles onto a wild-type background.

### Direct fitness competition in AYE

To determine the relative competitive fitness of *L. pneumophila* before and after evolution in our model, we constructed strains that could be co-cultured in AYE and distinguished by the presence or absence of GFP. The GFP-negative control vector pMMB207c and the GFP-positive pXDC31 are distinguished by the insertion of a GFP coding sequence whose expression is driven by pTac and ultimately induced by IPTG. Both plasmids were introduced into the wild-type ancestor Philadelphia-1 and the derived lineages C-1 and HA-1 by electroporation, as previously described (64). A single isolate was collected from each electroporation to represent the fitness of the population in competition.

Strain pairs containing these two plasmids were separately grown overnight and normalized to an OD_600_ of 0.1 in AYE supplemented with chloramphenicol for use in a direct competition assay, described in brief below. Competitions were carried out under standard incubation conditions. For each replicate, 500 µL of each culture of the strain pair was combined at a 1:1 ratio. A 100-µL sample of this mixture was used to inoculate 900 µL AYE with chloramphenicol and adjusted to a final OD_600_ of 0.01 and incubated overnight. The overnight culture was diluted to an OD_600_ of 0.01 and used to continue the assay in the same way for a total of three transfers. CFU concentration for each timepoint was counted by serial dilution plating on AYE with chloramphenicol and IPTG. Population fractions were manually counted under a UV black light to distinguish between fluorescent and non-fluorescent colonies.

### Host cell infection

*Vermamoeba vermiformis* was used as a permissive host to assess the infectivity of *L. pneumophila*. The amoeba was routinely grown in 25 mL vented flasks (Sarstedt, Corning) at room temperature and passaged twice a week in modified PYNFH media with FBS and buffer. Amoeba populations were expanded in 75 mL flasks 3 days before infection and harvested by centrifugation at 200 × *g* for 10 min.

Host cells were normalized in modified PYNFH without FBS or buffer at 5 × 10^5^ cells/mL, and 1 mL was added to each well of a 24-well cell culture plate. Infectious bacteria were collected from fresh colonies on CYE plates and normalized in Fraquil to an OD of OD_600_ 0.01 (approximately 10^7^ CFU/mL). To start infections with an MOI of 0.1, 5 µL bacterial suspension was added to each well, and the initial CFU count was conducted on the gently mixed supernatant. Growth was carried out for 3 days at 37°C with CFU counts performed daily.

### Western blotting

Protein samples for Western blotting were prepared using a modified version of the Bio-Rad electrophoresis guide (49). Briefly, *L. pneumophila* pellets were harvested by centrifugation from a 200-µL sample of overnight culture rinsed in Fraquil and normalized to an OD_600_ of 1. Pellets were homogenized by bath sonication (Cole-Parmer) on ice for 10 min after thorough suspension in 200 µL sample solubilization buffer. Samples were diluted with 250 µL 2× Laemmli sample buffer (BioRad) and held at room temperature for 20 min. The sample was centrifuged for 30 min, and the protein sample was collected from the resulting supernatant.

The proteins were migrated in hand-cast 12.5% polyacrylamide gels (BioRad) at 50 V for 30 min and 120 V for 90 min. Separated proteins were transferred onto polyvinylidene difluoride membranes (BioRad) at 25 V for 12 h at 4°C in preparation for immunoblotting and blocked using TBS wash buffer with 5% skim milk powder. Ab225994 (rabbit anti-*E*. *coli* HtpG; AbCam) was found to be cross-reactive to *L. pneumophila* HtpG and was used as the primary antibody at a 1:5,000 dilution, along with rabbit anti-IcdH (Sigma-Aldrich) (1:10,000) as a loading control. Primary incubation was carried out for 2 h, followed by rinsing and blotting with a 1:5,000 goat anti-rabbit HRP (Sigma-Aldrich) for 1 h. Bands were developed using an ECL Prime Western Blotting Detection Reagent (Cytiva BioSciences) and imaged using a ChemiDoc MP Imaging System.

## Data Availability

Raw read data from sequenced isolates collected after 30 and 70 passages have been deposited to the National Center for Biotechnology Availability’s sequence read archive and are available under BioProject Accession number PRJNA956983.

## SUPPLEMENTAL MATERIAL

Table S1 aem.00666-23-s0001.xlsx Summary of mutations identified by Breseq after 70 passages.

Table S2 aem.00666-23-s0002.xlsx *Legionella pneumophila* genome references used during the construction of gene alignments in environmental and clinical isolates.

Table S3 aem.00666-23-s0003.xlsx Local alignments of amino acid or nucleotide sequences in environmental and clinical isolates to search for the putative heat-shock resistance alleles developed in the laboratory model.

Table S4 aem.00666-23-s0004.xlsx Breseq output of mutations after 30 passages, used to support our conclusion of contamination in HA-4.

ASM does not own the copyrights to Supplemental Material that may be linked to, or accessed through, an article. The authors have granted ASM a non-exclusive, world-wide license to publish the Supplemental Material files. Please contact the corresponding author directly for reuse.
